# Mucopolysaccharidosis Type I Disease Prevalence Among Patients With Idiopathic Short Stature in Saudi Arabia: Protocol for a Multicenter Cross-sectional Study

**DOI:** 10.2196/28619

**Published:** 2021-08-31

**Authors:** Danyah Alsafadi, Aly Ezzat, Fatima Altamimi, Marwan ElBagoury, Mohammed Olfat, Mohammed Saleh, Sherif Roushdy, Yahia Aktham

**Affiliations:** 1 Al Aziziah Maternity and Children Hospital Jeddah Saudi Arabia; 2 Medical Affairs Department Sanofi-Genzyme Jeddah Saudi Arabia; 3 Yamamah General Hospital Riyadh Saudi Arabia; 4 Maternity and Children Hospital Madinah Saudi Arabia; 5 King Fahad Medical City Riyadh Saudi Arabia

**Keywords:** mucopolysaccharidosis, lysosomal storage disorders, epidemiology, Saudi Arabia

## Abstract

**Background:**

Since the underlying cause of idiopathic short stature can indeed be undiagnosed mucopolysaccharidosis type I, it is critical to identify patients with mucopolysaccharidosis type I among screened patients with idiopathic short stature.

**Objective:**

The primary objective of this study is to determine the prevalence of mucopolysaccharidosis type I disease in a high-risk group (ie, patients with idiopathic short stature).

**Methods:**

We plan to perform a multicenter, cross-sectional screening study to primarily assess the prevalence of mucopolysaccharidosis type I disease in patients with idiopathic short stature. All eligible patients will be tested after obtaining written informed consent from their parents and guardians. Eligible patients will be recruited over 18 months from specialty care centers for pediatrics and genetics.

**Results:**

This protocol was approved by the Institutional Review Board of King Fahd Medical City and funded by Sanofi Genzyme Saudi Arabia. We expect to collect data from ≥800 patients, as determined by our sample size calculation.

**Conclusions:**

Saudi Arabia is the largest country in the Arabian Peninsula; it has a population of more than 28 million people. To date, there are no reliable data regarding the incidence and prevalence of mucopolysaccharidosis type I in Saudi Arabia; therefore, future multicenter studies will be needed. Further, the prevalence of an attenuated form of mucopolysaccharidosis type I is largely underestimated in Saudi Arabia due to the absence of an effective newborn screening program. Therefore, the implementation of a nationwide newborn screening program is essential for the accurate estimation of the burden of mucopolysaccharidosis and the early diagnosis of patients.

**International Registered Report Identifier (IRRID):**

PRR1-10.2196/28619

## Introduction

Mucopolysaccharidoses are a group of chronic progressive disorders with multisystem affection and a fatal disease course; each disorder results from the significant deficiency of 1 of the 10 known enzymes that contribute to the lysosomal degradation of glycosaminoglycans [1]. Mucopolysaccharidosis type I is a common form of mucopolysaccharidosis; it accounts for nearly 15% of all mucopolysaccharidosis cases and occurs secondarily to a deficiency in α-L-iduronidase enzyme activity and the subsequent intracellular accumulation of dermatan and heparan sulfate [2]. Mucopolysaccharidosis type I is an autosomal recessive disorder with a progressive course and multisystem involvement [3]. The condition is associated with a wide range of phenotypic and clinical features. However, there are three distinct phenotypes of mucopolysaccharidosis type I—the Hurler, Hurler-Scheie, and Scheie syndromes (Scheie syndrome is the most severe form of mucopolysaccharidosis type I) [4]. Patients with mucopolysaccharidosis type I can present with characteristic facial features, cognitive and neurological impairments, hearing impairments, eye problems, cardiomyopathy, heart failure, recurrent respiratory infections, acute and chronic liver failure, joint contractures, and cervical instability, and spinal stenosis [2]. Further, patients with mucopolysaccharidosis type I are at higher risk of morbidity and mortality during anesthesia and surgical interventions [5].

The diagnosis of mucopolysaccharidosis type I depends on the detection of glycosaminoglycans in urine and a significant deficiency in the activity of the α-L-iduronidase enzyme. Alongside biochemical analysis, molecular tests play a critical role in the identification of the genotype of mucopolysaccharidosis; knowing the genotype can aid in the identification of the phenotype, genetic counseling, and prenatal diagnosis [6]. With regard to mucopolysaccharidosis diagnosis, different methods are available for the early diagnosis of mucopolysaccharidosis type I. These methods are based on the detection of deficient enzyme activity via dried blood spot (DBS) punches. DBS punching is a blood sampling technique in which a drop of blood is dried and placed on filter paper (a DBS card) [7]. These blood samples can be shipped to central laboratories—those in the same country or those from abroad—to be analyzed by using different methods such as enzymatic assays and confirmatory molecular testing, as in our study. Conventional fluorometric methods are widely available techniques for the detection of enzymatic activity; however, they have limited value due to their inability to test multiple enzymes simultaneously [8]. Tandem mass spectrometry (MS/MS) methods, which quantify lysosomal enzyme activity, have exhibited high diagnostic accuracy for the detection of lysosomal storage disorders (LSDs) and have a high capacity for multiplex testing [9]. Recent reports have also introduced new, cheap, and feasible MS/MS-based methods for the mass detection of mucopolysaccharidosis type I [10,11].

All mucopolysaccharidosis types are characterized by musculoskeletal manifestations in the form of joint stiffness, reduced joint mobility, carpal tunnel syndrome, and bone abnormalities [12]. Idiopathic short stature is widely considered as the main feature of mucopolysaccharidosis type I [13]; in previous studies, children with mucopolysaccharidosis type I constantly had growth measure values that were below the normal percentiles for age and sex and independent of disease severity and the age of onset [14,15]. Since the underlying cause of idiopathic short stature can indeed be undiagnosed mucopolysaccharidosis type I, it is critical to identify patients with mucopolysaccharidosis type I among screened patients with idiopathic short stature. Therefore, the primary objective of our study is to determine the prevalence of mucopolysaccharidosis type I disease in a high-risk group (ie, patients with idiopathic short stature). The secondary objectives are to describe the demographic profiles and clinical characteristics of patients with mucopolysaccharidosis type I and to describe other comorbid conditions in patients with mucopolysaccharidosis type I.

## Methods

### Study Design and Participants

We plan to perform a national, multicenter, cross-sectional screening study in which an interventional diagnostic procedure (DBS enzymatic assays followed by confirmatory molecular testing) will be conducted for each patient who meets the inclusion criteria in the outpatient setting. The study will be noninterventional in terms of the therapeutic strategy. The primary aim is to assess the prevalence of mucopolysaccharidosis type I disease in patients with idiopathic short stature.

### Inclusion Criteria

Patients will be deemed eligible for the study if they meet the following criteria:

- Male and female children aged between 2 and 15 years with idiopathic short stature if the treating physician believes that the underlying cause of idiopathic short stature could be undiagnosed mucopolysaccharidosis type I
- Patients who are confirmed to have at least 1 symptom of mucopolysaccharidosis type I, including growth impairment, hepatosplenomegaly, claw hand, carpal tunnel syndrome, and skeletal involvement (eg, kyphoscoliosis, joint stiffness, joint limitation, hernia, or scoliosis or corneal clouding)

Idiopathic short stature will be defined as a height of more than 2 SDs below the corresponding average height for a given age and sex.

### Exclusion Criteria

Patients will be deemed ineligible for the study if they meet the following criteria:

- Patients who are already known to have mucopolysaccharidosis disease
- Patients with a confirmed growth hormone deficiency
- Patients with other known causes of short stature, such as endocrine, genetic, and organ system disorders
- Patients who have already undergone DBS enzymatic assay tests

All eligible patients will be tested after obtaining written informed consent from their parents and guardians.

Eligible patients will be recruited over 18 months from specialty care centers for pediatrics and genetics, which have a fair amount of experience with managing patients with idiopathic short stature and may have experience with clinical research.

### Evaluation Criteria

#### Main Evaluation Criteria

The main evaluation criterion will be the percentage of subjects with confirmed (low enzymatic activity based on the enzyme assay and positive molecular tests; ie, “genetic pathogenic mutation detected”) mucopolysaccharidosis type I disease.

#### Secondary Evaluation Criteria

The description of demographic profiles and patient characteristics will include age, gender, risk factors (eg, a family history of mucopolysaccharidosis disease and the ethnicity of patients with mucopolysaccharidosis I disease; risk factors will be compared to those of the rest of the screened population), and the frequency of comorbid conditions in patients with mucopolysaccharidosis type I disease.

### Sample Size Calculation and Sampling Technique

The primary objective of this study is to evaluate mucopolysaccharidosis type I prevalence in the high-risk group (patients with idiopathic short stature). According to the Saudi Arabia Demographics Profile 2018, about 26.1% of the Saudi population are aged under 15 years [16]. Moreover, El Mouzan et al [17] reported that the prevalence of moderate and severe short stature in Saudi male and female children is 24.8%, as shown in Table 1.

**Table 1:** The prevalence of short stature among children in Saudi Arabia.

Sex	Children with moderate short stature, %	Children with severe short stature, %
Male	11.3	1.8
Female	10.5	1.2

The expected number of people with short stature aged under 15 years in Saudi Arabia is about 2,193,698. According to Pedicelli et al [18], about 80% of short children have no history of low birth weight and length and no detectable pathologies (ie, idiopathic short stature). Therefore, we expected to find 1,754,958 patients with idiopathic short stature aged under 15 years in Saudi Arabia, given that the overall Saudi population size is 33,891,021 (based on the latest United Nations estimates).

The expected prevalence of mucopolysaccharidosis type I disease worldwide is 3.5 to 4.5 patients per 100,000 people among the overall population [3]. On the other hand, the expected prevalence of mucopolysaccharidosis type I in Saudi Arabia is 3.31 patients per 100,000 people (0.003%) [19]. We calculated a sample size of 720 patients with idiopathic short stature aged under 15 years and an acceptable absolute deviation (95% CI) of 0.04% between the sample rate and the population rate (precision rate) in Saudi Arabia. We expect to have a 10% dropout rate resulting from a lack of data. Therefore, 800 patients will be required for this study. The sample size was calculated by using StatsDirect Statistical Analysis Software (version 3.1.17; StatsDirect Ltd).

### Data Collection

Following an initial screening test, all potentially eligible patients will undergo anthropometric examinations to confirm the diagnosis of short stature. The following equations will be used:

Stature of females = (maternal stature + paternal stature – 12.5 cm)/2 **(1)**

Stature of males = (maternal stature + paternal stature + 12.5 cm)/2 **(2)**

The following data will be collected from each patient: sociodemographic profiles; findings from the physical examination; anthropometric examination findings, including height, weight, BMI, and body circumference (waist, hip, and limb circumferences); a history of comorbidities; a history of previous surgeries; a family history of inborn errors of metabolism; presenting symptoms; findings from routine laboratory investigations; a history of mucopolysaccharidosis treatment; and findings from enzymatic assay screening.

**Figure 1:**
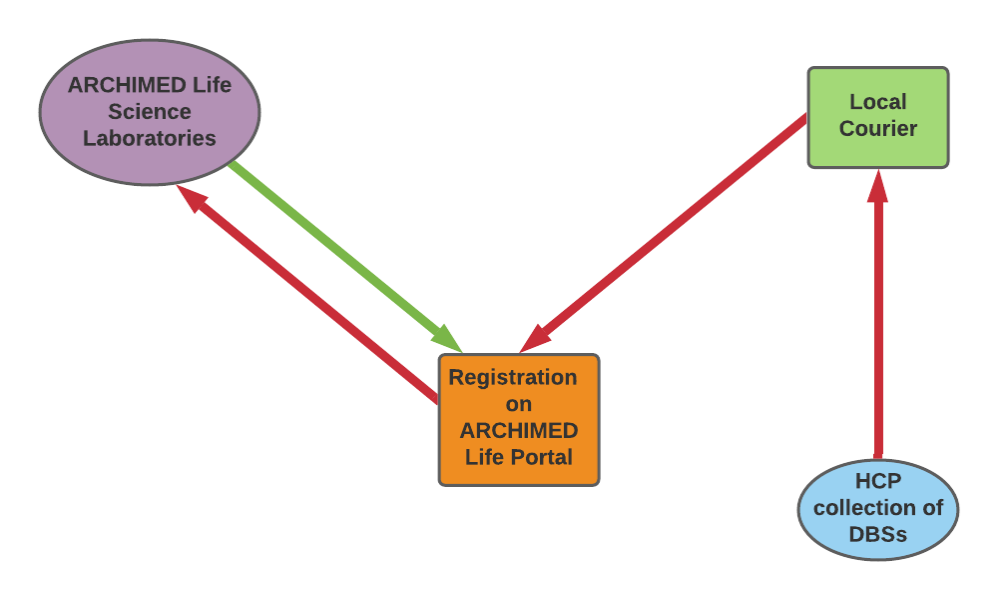
A flowchart depicting the logistic aspects of the study. Lab results will be uploaded on Arvado (ARCHIMED Life Sciences Laboratories) by the lab and will be available on each HCP account. DBS: dried blood spot; HCP: health care personnel.

A central laboratory will provide ARCHIMED Life Science Laboratories DBS and genetic test services to all sites participating in the study. All patient data (except the results of the investigation) will be collected during a single visit. Each enrolled patient will visit the investigators for a baseline visit. The investigators will complete the electronic case report form (eCRF) soon after the baseline visit. Upon receiving the test results, investigators will be required to report in the eCRF (within 5 days) that the results that will be provided to their respective patients. The general logistic aspects of the study are presented in Figure 1.

An independent contract research organization (CRO) will provide the study centers with the proper levels of access, grants, and privileges for the eCRFs, which will be filled by the investigators or the authorized designees according to the complete guidelines. Data entry screen development, validation rules programming, and the maintenance of the study database will be the responsibility of the independent CRO. The computerized handling of the data by the CRO may generate additional queries, which will be automatically identified via preprogrammed and tested validation rules. Validation rules will be detailed in the data validation plan. In addition to automatic validation rules, a manual and medical review of data may generate further queries; these will be uploaded to the system as well. Site staff will be responsible for resolving automatic and manual queries by confirming or modifying the data, which will be collected with the electronic data capture system. The data collection and validation procedures will be detailed in the operational study manual.

### Study End Points

The primary end point is the prevalence of mucopolysaccharidosis type I in the high-risk group (patients with idiopathic short stature), which will be confirmed by enzyme activity DBS testing. Other end points include a description of demographic profiles and patient characteristics and the frequency of comorbid conditions in patients with mucopolysaccharidosis type I.

### Statistical Methods

All data collected during the study will be analyzed with the appropriate descriptive analysis. Statistical analyses will be performed by using SPSS version 18 or higher (IBM Corporation). The prevalence of mucopolysaccharidosis type I will be described by using counts and percentages with 95% CIs. Other variables will be described by using means and SDs (for continuous variables) or counts (for categorical variables). Patients’ variables will be compared by using Mann-Whitney-Wilcoxon tests for continuous parameters and Chi-square tests for categorical parameters. A *P* value of less than .05 will be considered significant.

### Ethics Approval and Consent to Participate

The study’s protocol was approved by the ethics committee of King Fahd Medical City for participant hospitals (Aseer Central Hospital and Abha and Yamamah General Hospital in Riyadh). The study protocol was registered by the Institutional Review Board (IRB) of King Abdulaziz City for Science and Technology.

Written informed consent will be obtained from every eligible patient prior to the sample withdrawal.

This study will be conducted in accordance with the principles laid out by the 18th World Medical Assembly (1964 Helsinki Declaration) and all their subsequent amendments.

The study will be conducted in accordance with the US and European guidelines for Good Epidemiology Practice. All necessary regulatory submissions (eg, IRB and independent ethics committee) were performed in accordance with Saudi Arabia’s local regulations, including the local data protection regulations.

### Consent for Publication

If case reports are presented in the study, consent for publication will be obtained from the subject of the case report. If children are the subject of the case report, consent for publication will be obtained from their parents and legal guardians.

### Availability of Data and Materials

Data sharing does not apply to this paper, as no data sets were generated or analyzed in this manuscript.

## Results

Our protocol was reviewed and approved by the IRB of King Fahd Medical City and funded by Sanofi Genzyme Saudi Arabia. According to our sample size calculation, we expect that at least 800 patients from different sites in Saudi Arabia will be enrolled in our study.

## Discussion

The early diagnosis of mucopolysaccharidosis followed by early treatment can have a huge impact on patients’ quality of life; previous reports have demonstrated that the early initiation of treatment is the most important factor for slowing the progression of the disease [2]. Enzyme replacement therapy, when delivered early in the disease process, has alleviated many systematic signs and symptoms of mucopolysaccharidosis. This is possibly due to the prevention of permanent tissue damage resulting from excessive substrate deposition [20]. Since many mucopolysaccharidosis type I symptoms are often unspecific, many patients, especially those with undiagnosed mucopolysaccharidosis type I or an attenuated form of the disease, are either misdiagnosed or diagnosed later in the course of the disease (ie, after precious time has been lost due to inappropriate treatment and after irreplaceable organ damage has occurred) [21]. At some point during the course of the disease, patients with mucopolysaccharidosis consult rheumatologists. As such, rheumatologists should be aware of clinical manifestations that raise the suspicion of a mucopolysaccharidosis diagnosis. Thus, we found it imperative to assess the prevalence of mucopolysaccharidosis type I among high-risk patients, such as pediatric patients with idiopathic short stature.

The added values of our screening program include (1) the determination of the actual prevalence of mucopolysaccharidosis type I among children with idiopathic short stature, (2) the early detection of potentially disabling conditions among Saudi children (these data will provide a chance for early treatment and better prognoses), and (3) the increased awareness of Saudi pediatricians and primary care physicians with regard to how and when to suspect a mucopolysaccharidosis diagnosis in children.

We chose to conduct this epidemiological study in Saudi Arabia due to the peculiar characteristics of this country, which is located in the Arabian Peninsula and has a population of more than 28 million people. Recent reports have noted a trend toward a higher incidence of genetic disorders among Arab countries when compared to those of other parts of the world. These incidence rates also apply to the incidence of mucopolysaccharidosis type I. Recent global data show that the overall birth prevalence rate of mucopolysaccharidosis ranges from 1.04 to 4.8 infants per 100,000 live births. Additionally, reports from Europe show that the incidence rate of mucopolysaccharidosis is 1.56 infants per 100,000 live births [3]. This incidence rate is quite similar to the mucopolysaccharidosis incidence rates of Japan and other East Asian countries that were reported during the same period [22-24]. On the other hand, reports from Saudi Arabia show that the country has a higher incidence of mucopolysaccharidosis compared to those in the abovementioned reports. The first retrospective study from Saudi Arabia on this topic reported that the combined incidence rate of mucopolysaccharidosis type I and mucopolysaccharidosis type IV was 3.62 infants per 100,000 live births, and each disease type accounted for 21% of all mucopolysaccharidosis cases. Moreover, the birth prevalence of mucopolysaccharidosis III is 1.8 infants per 100,000 live births (11% of total cases) [25]. In addition, Al-Sannaa and colleagues [19] reported that the incidence rate of mucopolysaccharidosis diseases was 14 infants per 100,000 live births; with mucopolysaccharidosis type VI accounted for most of the mucopolysaccharidosis cases. A 13-year retrospective chart review reported an incidence rate of 14 infants per 100,000 live birth [26]. The incidence of mucopolysaccharidosis in Saudi Arabia appears to be even higher than those reported by other Arab countries; a previous report from Tunisia reported a mucopolysaccharidosis birth prevalence of 2.27 infants per 100,000 live births [27]. These high figures were postulated to stem from the higher incidence of congenital and genetic diseases in Arab countries compared to those of other parts of the world [28]; high consanguinity rates, which reach up to 60% in some regions; the high prevalence of hemoglobinopathies and metabolic disorders; relatively high maternal and parental ages; and the lack of proper genetic screening [28-30].

With regard to mucopolysaccharidosis diagnosis, different methods are available for the early diagnosis of mucopolysaccharidosis type I. These methods are based on the detection of deficient enzyme activity via DBS punches. Conventional fluorometric methods are widely available techniques for the detection of enzymatic activity; however, they have limited value due to their inability to test multiple enzymes simultaneously [8]. MS/MS methods, which quantify lysosomal enzyme activity, have exhibited high diagnostic accuracy for the detection of LSDs and have a high capacity for multiplex testing [9]. Recent reports have also introduced new, cheap, and feasible MS/MS-based methods for the mass detection of mucopolysaccharidosis type I [10,11]. Such advances in the diagnostic methods have encouraged other researchers to conduct several mucopolysaccharidosis type I neonatal screening programs; these researchers aim to evaluate the utility of mucopolysaccharidosis type I neonatal screening to determine whether such screening should be included in primary screening programs. From 2008 to 2013, a pilot screening program for mucopolysaccharidosis type I was conducted for 35,286 newborns from Taiwan. Only 2 neonates had a confirmed diagnosis of mucopolysaccharidosis type I. The rate of mucopolysaccharidosis type I incidence in Taiwan that was estimated from the results of this program was about 1:17,643 [31]. In the United States, several states have conducted pilot mucopolysaccharidosis screening programs. In a comprehensive program for LSDs that was conducted in Missouri, a multiplexing digital microfluidic fluorometric enzymatic assay was used to detect Pompe disease, Fabry disease, Gaucher disease, and mucopolysaccharidosis type I in 2013. Of the 43,701 screened newborns, 3 had a confirmed diagnosis of mucopolysaccharidosis type I, and 7 had pseudodeficiencies. In this Missouri program, the rate of mucopolysaccharidosis type I incidence (1:14,567) was similar to the incidence rate reported in a previous pilot study conducted in Taiwan (1:17,643) [32]. In Saudi Arabia, a national newborn screening program was established in 2005. This program covers inborn errors of metabolism, endocrine disorders, congenital heart defects, and hearing loss [33]. A recent 7-year retrospective study of 139 hospitals reported a higher rate of inborn errors of metabolism in Saudi Arabia compared to those in other parts of the world [34]. However, the inclusion of LSDs, including mucopolysaccharidosis type I, in Saudi Arabia’s newborn screening program has not yet been discussed.

## Abbreviations

CRO: contract research organization
DBS: dried blood spot
eCRF: electronic case report form
IRB: Institutional Review Board
LSD: lysosomal storage disorder
MS/MS: Tandem mass spectrometry
